# Systematic Review and Transcriptomic Meta-analysis of Environmental Enrichment Reveal Core Molecular Programs of Brain Plasticity

**DOI:** 10.1007/s12035-026-06133-y

**Published:** 2026-08-26

**Authors:** Marcelina Kurowska, Federico Miozzo, Robert Schroeder, Magdalena A. Machnicka, Rocío Pérez-González, Karine Merienne, André Fischer, Angel Barco, Anne-Laurence Boutillier, Bartek Wilczyński

**Affiliations:** 1 Faculty of Mathematics, Informatics and Mechanics, University of Warsaw, 00-927 Warsaw, Poland; 2 Instituto de Neurociencias, Universidad Miguel Hernández - Consejo Superior de Investigaciones Científicas, Av. Santiago Ramón y Cajal S/N. Sant Joan d’Alacant, 03550 Alicante, Spain; 3 Department for Epigenetics and Systems Medicine in Neurodegenerative Diseases, German Center for Neurodegenerative Diseases (DZNE), Von Siebold Str. 3 A, 37077 Göttingen, Germany; 4 Instituto de Investigación Sanitaria y Biomédica de Alicante (ISABIAL), 03010, Alicante, Spain; 5 Laboratoire de Neuroscience Cognitives Et Adaptatives (LNCA), UMR7364 CNRS, University of Strasbourg, 67000 Strasbourg, France; 6 Cluster of Excellence “Multiscale Bioimaging: From Molecular Machines to Networks of Excitable Cells” (MBExC), University of Göttingen, Von Siebold Str. 3 A, 37077 Göttingen, Germany; 7 German Center for Cardiovascular Diseases (DZKH), Göttingen, Von Siebold Str. 3 A, 37077 Göttingen, Germany; 8 Department for Psychiatry and Psychotherapy, University Medical Center of Göttingen, Georg-August University, Von Siebold Str. 3 A, 37077 Göttingen, Germany; 9 Centro de Investigación en Red-Enfermedades Neurodegenerativas (CIBERNED), 28031 Madrid, Spain

**Keywords:** Enriched environment, Meta-analysis, Transcriptomics, Cognition, Synapse

## Abstract

Environmental enrichment (EE) paradigms in rodents have long demonstrated that enhanced sensory, cognitive, social, and motor stimulation positively impacts brain function, improving learning, memory, and neuroplasticity. These effects have significant implications for understanding cognitive development and mitigating cognitive decline and brain aging. While numerous transcriptomic studies have explored EE-induced molecular changes, a unified view of the genes and pathways consistently modulated remains lacking. To address this gap, we performed a systematic review and meta-analysis. We conducted a comprehensive PubMed search for all studies published up to February 2025 that matched all the following inclusion criteria: (1) employed EE paradigms; (2) were conducted on rodents; (3) utilized genome-wide transcriptomic methods; (4) examined brain regions or neuronal populations. The 323 retrieved articles were manually screened for relevance to the study aims and data availability. Datasets from 20 eligible RNA-seq reports were reprocessed using a unified analysis pipeline and subjected to a meta-analysis with three complementary statistical methods. Despite considerable heterogeneity across studies, our integrative analysis identified consistent gene expression signatures linked to synaptic function, plasticity and their transcriptional regulation. In particular, our findings highlight the upregulation of the activity-dependent transcriptional program, including *Fos* and *Jun* family members. These molecular insights advance our understanding of how EE impacts on neuronal and behavioral outcomes, and may inform therapeutic strategies aimed at replicating or enhancing EE benefits. To promote open science and foster further research, we developed an accessible web application, mEEtaBrain, that enables the neuroscience community to navigate and interrogate our meta-analysis results. Substantial methodological heterogeneity across source studies increased variability in the meta-analysis outcomes. The use of stressors or disease models, particularly in rat studies, introduced a major confounding factor and limited reliable interspecies comparison. Overall, the studies exhibited a low to moderate risk of bias.

**Supplementary Information:**

The online version contains supplementary material available at 10.1007/s12035-026-06133-y.

## Introduction

The development of the mammalian brain is guided by complex genetic and epigenetic programs that establish most of its structural framework before birth. Yet, the continuous refinement of neural circuits essential for normal cognitive and behavioral function depends heavily on inputs from the environment during childhood and adulthood [1]. There is broad consensus that a stimulating lifestyle, combining physical activity, rich social interactions, and intellectual stimulation, can significantly enhance cognitive development and reduce the risk of cognitive decline in elderly humans [2, 3].

Enriched environments (EE) have been widely used in rodent models to investigate how external stimulation influences brain function and to uncover the underlying neurobiological mechanisms. In these paradigms, rodents are housed in settings that offer enhanced sensory, cognitive, social, and motor stimulation relative to standard conditions. Typically, EE involves keeping animals in large groups within an ample space, with opportunities for voluntary exercise and exploration of novel objects, where toys and running wheels are regularly replaced to maintain novelty. Decades of research have demonstrated that EE can enhance learning and memory performances, both in wild-type animals and in a range of neuronal dysfunction models [4–7], including Huntington’s Disease [8, 9], Alzheimer’s Disease [10–13], dementia [14], brain injury [15, 16], and intellectual disability disorders [17, 18]. Investigation of the associated neuronal processes revealed that EE stimulates hippocampal neurogenesis [19–21], increases spine density, dendritic length, and dendritic complexity in hippocampus and cortex regions [17, 22–24], improves neurotransmitter function [25, 26] and neurotrophic factors expression [27–29].

Building on this evidence from rodent studies, clinical occupational therapies and related approaches have gained support as potential interventions for a broad range of human conditions [30, 31], from neurodevelopmental disorders in children [32] to cognitive deterioration with aging [33]. Moreover, these studies sparked great interest in uncovering the precise molecular mechanisms by which EE improves brain function, aiming to identify novel therapeutic targets which could mimic or potentiate EE benefits. Transcriptional and epigenetic processes are well-established mediators between environmental stimuli and genomic regulation [34, 35], and play a pivotal role in orchestrating synaptic plasticity processes that underlie neuronal function and memory [36–40]. These mechanisms are thus appealing candidates for translating EE lasting effect into behavioral and cognitive improvements, and their understanding could be key to developing novel therapeutic interventions. However, despite the wealth of genome-wide studies on animals housed in EE over the past two decades, little effort has been made to integrate their findings, and no clear consensus has emerged on the genes and pathways regulated by EE in the brain.

To uncover consistent gene expression signatures associated with EE across multiple studies, we performed a systematic literature screening followed by meta-analysis of 20 independent brain transcriptomic studies from rodents exposed to EE. Despite substantial heterogeneity in gene expression profiles across studies, our integrative analysis reliably identified a set of reproducible transcriptional features of EE. These findings offer molecular insights into EE synaptic and behavioral effects, and may guide the development of targeted strategies aimed at mimicking or enhancing its cognitive benefits. To facilitate broader exploration of our meta-analysis by the neuroscience community, we also developed mEEtaBrain (http://regulomics.mimuw.edu.pl/mEEtaBrain/), a freely accessible, user-friendly web application.

## Results

### Compilation of Brain Transcriptomic Datasets from EE Studies

To conduct our meta-analysis, we aimed to collect all transcriptomic studies which met the following four criteria: (1) employed EE paradigms; (2) used rodents as the animal model, given the extensive EE literature for these species; (3) utilized genome-wide methods to profile gene expression, to ensure an unbiased, discovery-driven approach rather than a hypothesis-driven one limited to preselected genes; (4) examined brain regions or purified neuronal populations as tissue/cell-type of interest. To this end, we queried PubMed using a search string designed to capture all four aspects (see Methods for the full query and dataset compilation details). This search yielded a total of 323 peer-reviewed studies.

**Fig. 1:**
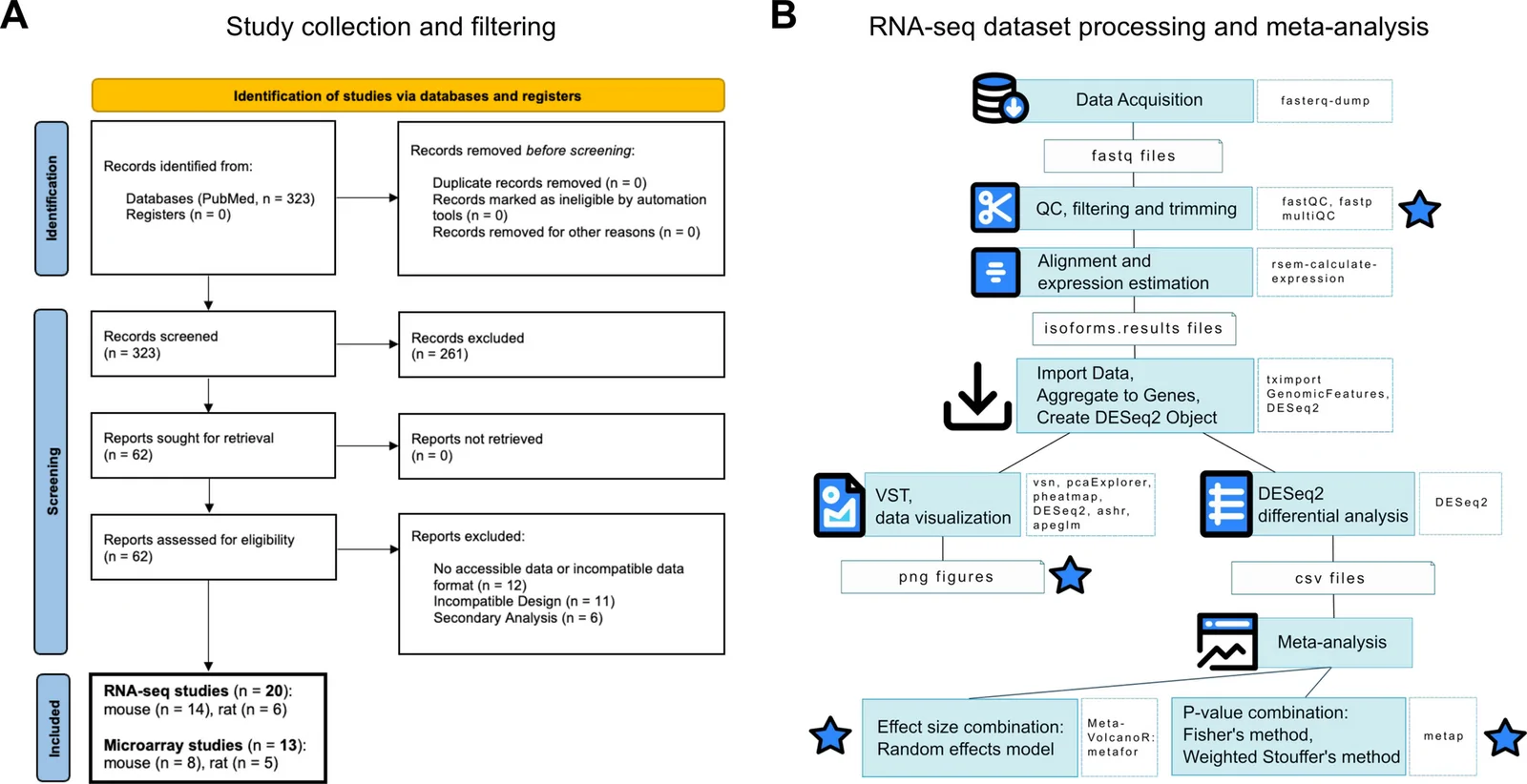
Overview of dataset collection and meta-analysis workflow. **A** Workflow of transcriptomic studies acquisition and filtering. **B** Pipeline of RNA-seq dataset processing and meta-analysis. A star indicates the features that can be explored in the online app

We then manually screened the abstracts to identify only those which fully satisfied the four inclusion criteria mentioned above. After this initial filtering, we retained 36 RNA-seq studies, including one using SAGE-seq, and 26 microarray studies. A second screen was performed based on data availability. At the end of our screening workflow (Fig. 1A), we retained 20 RNA-seq [41–60] and 13 microarray studies [13, 18, 26, 61–70]. More details about the selecting criteria can be found in the methods section.

The majority of these datasets was generated from mouse experiments (14 out of 20 for RNA-seq, 8 out of 13 for microarrays). These studies displayed large heterogeneity in both the EE paradigms used and the biological materials profiled, which included hippocampus or its subregions (17 studies), various cortical areas (9), striatum or its subregions (4), amygdala (2), brain stem (1), or specific neuronal populations purified from these regions. It should be noted that some papers applied EE in the context of brain pathologies or stressors; in such studies, we focused on EE-associated changes rather than in the disease-related changes. Species, genetic background, age, sex, EE paradigm, CNS region, method of transcriptomic profiling, bias assessment, and literature reference for these datasets can be found in Supp. Table 1 and 2, whereas Supp. Table 3 and 4 illustrate EE paradigm conditions and duration.

### Meta-analysis of EE Transcriptomic Data Identifies Genes Consistently Modulated by EE

To ensure the robustness of the meta-analysis, we retrieved raw data from public repositories and re-analyzed all RNA-seq datasets using a unified pipeline (Fig. 1B). After quality control, filtering, and trimming, reads were quantified, aggregated to the gene level, visually assessed via PCA and clustering, and finally analyzed for differential expression. Overall, this standardized workflow allowed for consistent processing across datasets, providing a reliable foundation for downstream meta-analytic integration.

**Fig. 2:**
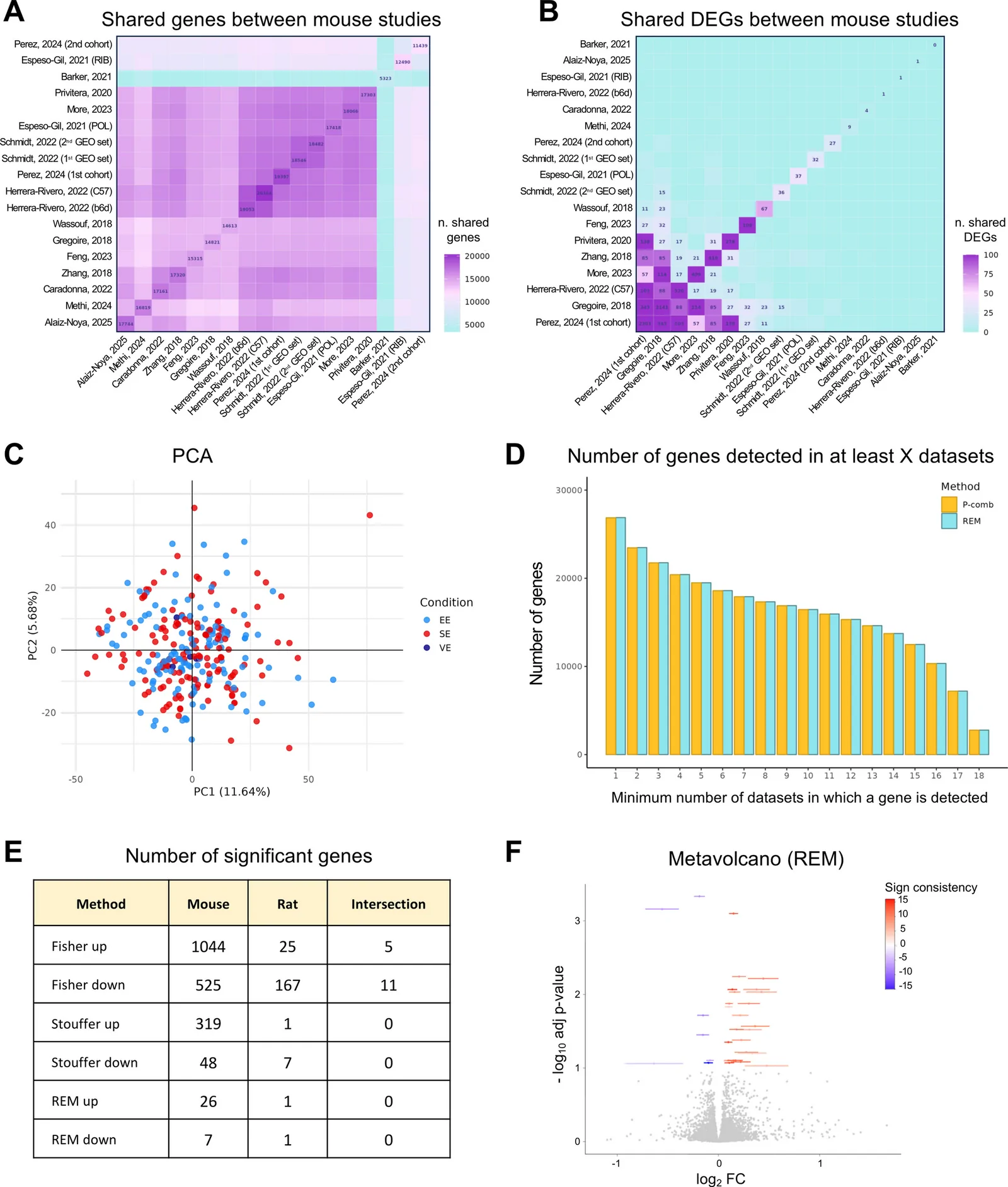
Overview of mouse RNA-seq studies and meta-analysis. **A** Heatmap showing the number of detected genes shared between pairs of mouse RNA-seq studies. **B** Heatmap showing the number of DEGs shared between pairs of mouse RNA-seq studies. **C** PCA of mouse RNA-seq datasets. EE, enriched environment (red); SE, standard environment (blue); VE, voluntary exercise (dark blue). *N* = 36 (EE), 37 (SE), 4 (VE). **D** Number of genes for which meta-analysis statistics were computed plotted against the minimum number of studies in which each gene was detected. **E** Number of significant genes up- or down-regulated in EE vs SE identified by meta-analysis of mouse and rat datasets, and their intersection. A minimum of 6 (mouse) and 2 (rat) datasets with detected gene expression values was required for a gene to be included in the corresponding meta-analysis. REM, Fisher and weighted Stouffer, adj *p*-value < 0.01. **F** Volcano plot from REM meta-analysis of mouse RNA-seq studies. A minimum of 6 datasets with detected gene expression values was required for a gene to be included. Error bars represent 95% confidence intervals. Genes significantly upregulated and downregulated under EE are colored in red and blue, respectively (adj *p*-value < 0.01)

Regarding RNA-seq data, most mouse studies detected more than 14,000 genes and shared over 10,000, providing a solid basis for data integration, with only one dataset [41] containing substantially fewer genes (~ 5000) (Fig. 2A). Similarly, all rat studies shared over 11,000 detected genes (Supp. Fig. S1A). Pairwise intersection of differentially expressed genes (DEGs) between EE and standard environment (SE) revealed modest overlap across studies (Fig. 2B and Supp. Fig. S1B). In agreement, principal component analysis (PCA) showed no obvious clustering of EE and SE samples in either mouse or rat datasets (Fig. 2C and Supp. Fig. S1C), suggesting that EE-induced transcriptional changes are moderate in magnitude and number of genes affected and likely masked by inter-study variability.

Under these conditions, simply intersecting DEGs from individual datasets is likely to yield poor results, as it relies on arbitrary thresholds to include or exclude genes in each study. To overcome this limitation and capture subtle but consistent expression changes shared among datasets, we implemented a meta-analysis. Specifically, we used two complementary approaches [71] (Fig. 1B):

i. *p-value combination methods*, including Fisher’s and weighted Stouffer’s methods, which integrate *p*-values of individual analysis into a single combined *p*-value per gene, to assess overall statistical significance across studies
ii. the *random effect model (REM)*, which combines effect sizes to derive a combined fold change per gene, to account for the magnitude and direction of gene expression changes.

This dual approach increased our ability to detect robust gene expression patterns associated with EE. Both methods generated meta-analysis statistics for over 10,000 genes, which were detected in at least 16 of the 18 mouse studies and in all rat datasets (Fig. 2D and Supp. Fig. S1D). Depending on the approach and parameters used, our meta-analysis revealed hundreds to thousands of significantly affected genes across mouse datasets (Fig. 2E and Supp. Tables 5–7), as illustrated by the volcano plot for REM method (Fig. 2F). One-study-out sensitivity analysis confirmed the robustness of our meta-analysis approaches (Supp. Fig. S2A–C). Notably, irrespective of the method used, upregulated genes largely outnumbered downregulated ones, suggesting that EE primarily enhances rather than represses gene expression. In contrast, far fewer significant genes were detected in rat studies, and inter-species overlap was minimal (Fig. 2E). This was likely due to the limited number of available rat datasets, and substantial confounding factors introduced by the use of addiction or brain disease models in the majority of rat studies (Supp. Table 2).

To facilitate exploration of the data, all resulting lists of EE-dependent genes can be interactively accessed through mEEtaBrain, a dedicated app which allows users to dynamically adjust meta-analysis methods and settings, explore integrated results, perform custom gene queries, and generate and download interactive plots and GO analysis.

To estimate heterogeneity across our datasets, we calculated the *I*^2^ statistics for each gene in the REM analysis and performed Cochran’s QE test for the same genes. For most mouse and rat genes, *I*^2^ values were low, indicating that the majority of the observed variance was attributable to random sampling error rather than between-study heterogeneity (Supp. Fig. S3A, B). Importantly, genes identified as significant by REM analysis showed little overlap with genes exhibiting significant heterogeneity according to Cochran’s QE test or high *I*^2^ values (Supp. Fig. S3C, D). These results suggest that the main transcriptional signatures identified in our meta-analysis were not driven by substantial between-study heterogeneity. Furthermore, we performed a meta-regression analysis that included potential confounding variables as moderators such as brain region, sex, pathology/stress condition, and EE duration. The meta-regression indicated that these factors explained only a limited proportion of the variability in gene-level effect sizes, suggesting that the core transcriptional signatures identified in our meta-analysis are robust across these differences in experimental conditions. This was particularly evident among genes identified as significant by REM analysis (Supp. Fig. S3E, F). EE duration was the only moderator associated with a comparatively larger number of genes. However, these genes did not exhibit larger overall effect-size estimates than genes without significant moderator effects, and most were identified as significant by Fisher’s method (Supp. Fig. S3G). Overall, the examined sources of heterogeneity did not appear to systematically influence the estimated effects of EE for most genes.

Regarding microarray studies, raw data were unavailable for most datasets. Only lists of DEGs were provided, occasionally with associated fold changes. For genes not included in these lists, it was unclear whether they could not be detected or simply were not differentially expressed, making it impossible to assess their relative expression between EE and SE. Due to these limitations, a formal meta-analysis was not feasible. To still extract meaningful insight from these datasets, we identified DEGs consistently reported across combined mouse and rat microarray studies. Pairwise comparisons of DEG lists revealed limited overlap (Supp. Fig. S4A), with only 12 genes shared by at least three studies (*Arc*, *Bdnf*, *Btg2*, *Dlg4*, *Dnmt1*, *Dusp1*, *Egr1*, *Egr2*, *Egr4*, *Homer1*, *Junb*, *Nr4a1*) (Supp. Fig. S4B and Supp. Table 8). The intersection of the gene lists obtained with the three different RNA-seq meta-analysis methods and from microarray datasets revealed substantial overlaps (Supp. Fig. S4C). As expected, Fisher’s method identified a larger number of DEGs, consistent with its lower stringency compared to Stouffer’s and REM [72].

### EE Modulates Key Genes and Complexes Operating at the Synapse

**Fig. 3:**
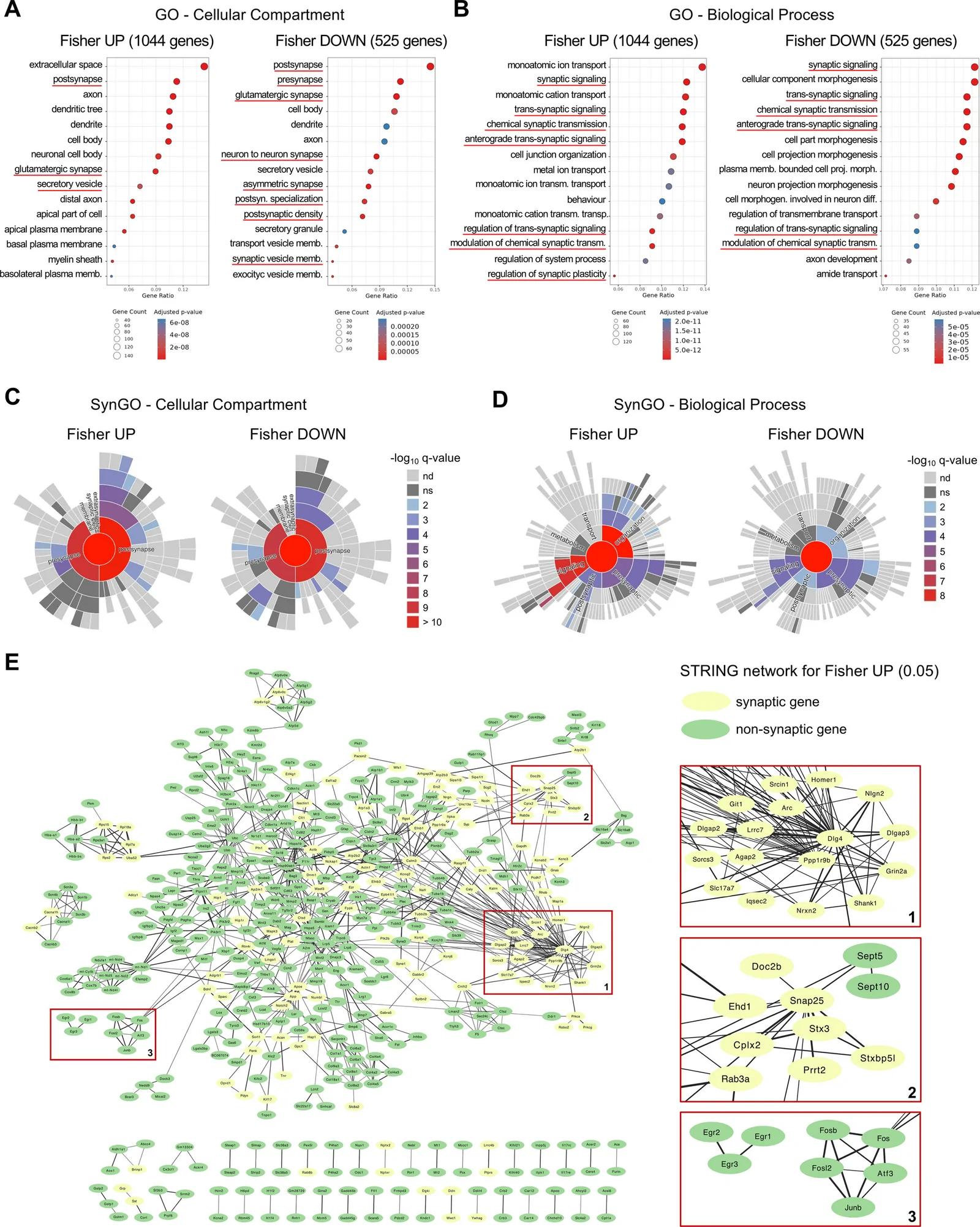
EE modulates key genes and complexes operating at the synapse. **A**, **B** Bubble plots illustrating Gene Ontology (GO) enrichment analysis for upregulated and downregulated genes obtained with Fisher’s method. A minimum of six datasets with detected gene expression values was required for a gene to be included. The 15 most significant GO terms for cellular compartment (**A**) and biological process (**B**) are shown. The bubble size and color indicate the number of genes associated to the term and the adjusted *p*-value, respectively. GO terms related to synapse are underlined in red. **C**, **D** SynGO analysis of cellular compartment (**C**) and biological process (**D**) for upregulated and downregulated genes obtained with Fisher’s method. **E** Protein–protein interaction network of the upregulated genes identified with Fisher’s method (adj *p*-value < 0.01). Network analysis was performed using STRING (physical subnetwork; cut-off for combined interaction score: 0.4) and visualized with Cytoscape. Edge thickness indicates the strength of data support. Only proteins displaying at least one interaction are shown. Side panels (1–3) are zoom-in of selected clusters within the network

To gain an unbiased insight into the biological processes modulated by EE, we performed gene ontology (GO) enrichment analysis on the genes significantly up- and downregulated by EE. The results illustrated in Fig. 3 are based on Fisher’s method meta-analysis (adj *p*-value < 0.01), which yielded a broader set of significant genes well suited for enrichment analysis. Alternative statistical thresholds and methods can be explored by users through the web app. GO cellular compartment analysis revealed a very strong enrichment for neuronal and, specifically, synaptic structures, as well as for terms related to extracellular space and membranes, both across up- and downregulated genes (Fig. 3A and Supp. Table 9). Consistently, biological processes GO categories pointed to synaptic transmission, signaling and plasticity (Fig. 3A and Supp. Table 9), while molecular function terms were enriched for transmembrane transporters and ion channel activity (Supp. Fig. S5A and Supp. Table 9). Analysis with the manually curated, synapse-focused SynGO resource confirmed high enrichment of synaptic genes across both presynaptic and postsynaptic compartments (Fig. 3C) and spanning multiple synaptic processes (Fig. 3D), including 188 upregulated and 120 downregulated genes. In particular, genes related to synapse organization and synaptic signaling were especially enriched among EE-upregulated genes, including the postsynaptic scaffolds *Dlg4*, *Ppp1r9b*, *Homer1*, *Shank1*, *Dlgap3*, the ion channels *Grin2a*, *Gabra5*, *Kcnj6*, the synaptic adhesion molecules *Nectin1*, *Nlgn2*, *Nrxn2*, *Pcdh7*, *Pcdh8*, *Igsf9*, *Robo2*, and multiple actors of the synaptic vesicle cycle such as *Snap25*, *Unc13a*, *Stx3*, *Doc2b*, *Prrt2*, *Stxbp5l*, *Syp*, and *Rab3a* (Supp. Table 10). Together, these analyses indicate that EE influences neuronal transcriptomes and broadly modulates genes involved in synaptic structure, function, and signaling.

To better understand the functional relationship among significant EE-upregulated genes, we conducted a protein–protein interaction analysis of their gene products using the STRING database. The resulting network displayed significantly more interactions than expected by chance, indicating strong enrichment in physical connectivity (*p*-value = 10^–16^) (Fig. 3E and Supp. Table 11). Among the top three hubs of the network, two were well-established scaffold proteins of the postsynaptic density (PSD): PSD-95 and Spinophilin, encoded by the genes *Dlg4* and *Ppp1r9b*, respectively. PSD-95 is a master organizer of excitatory synapse architecture. By anchoring NMDA and AMPA receptors and linking them to signaling complexes and the actin cytoskeleton, PSD-95 plays a central role in synapse stabilization, receptor clustering, and activity-dependent synaptic plasticity [73, 74]. Spinophilin targets protein phosphatase 1 (PP1), a key regulator of synaptic plasticity, to specific substrates, particularly glutamate receptors, thereby modulating AMPAR and NMDAR function and regulating synaptic efficacy and spine morphology [75–77]. The identification of *Dlg4* and *Ppp1r9b* and their associated protein network among EE-modulated genes (Fig. 3E, zoomed panel 1) highlights PSD and dendritic spines as critical neuronal compartments affected by EE.

We also found multiple core components (*Snap25*, *Stx3*) and modulators (*Cplx2*, *Stxbp5l*, *Doc2b*, *Prrt2*, *Rab3a*) of the SNARE complex (soluble NSF attachment protein receptor complex) (Fig. 3E, zoomed panel 2). The SNARE complex is essential for neurotransmitter release, driving synaptic vesicle fusion with the presynaptic membrane [78, 79]. It also mediates postsynaptic exocytosis and plasticity, including long-term potentiation (LTP) and spine growth [80–82]. The enrichment of SNARE complex genes in our meta-analysis further supports the notion that EE modulates genes involved in the dynamic regulation of synaptic function.

### EE Enhances the Activity-dependent Transcriptional Program

The diverse motor, social, and cognitive stimuli provided by EE are thought to activate neuronal circuits responsive to such inputs across multiple brain areas [6]. We reasoned that, if our meta-analysis reliably captures transcriptional features of EE, it should reveal a tonic increase in the expression of Immediate Early Genes (IEGs). These genes are rapidly induced in response to neuronal activation, and many of them encode transcription factors believed to play key roles in the transcriptional regulation of experience-dependent synaptic plasticity [83]. Indeed, among the non-synaptic proteins identified in our network, we found the AP-1 transcriptional complex composed of activity-induced FOS and JUN family members (Fig. 3E, zoomed panel 3). AP-1 is a master regulator of activity-dependent gene expression [83] and directly controls multiple synaptic genes [54, 84]. Therefore, alongside other activity-induced transcription factors identified in our meta-analysis such as EGR 1–3, NPAS4 and NR4A1, AP-1 might likely contribute to the widespread transcriptional modulation of synaptic genes revealed by our GO analysis.

**Fig. 4:**
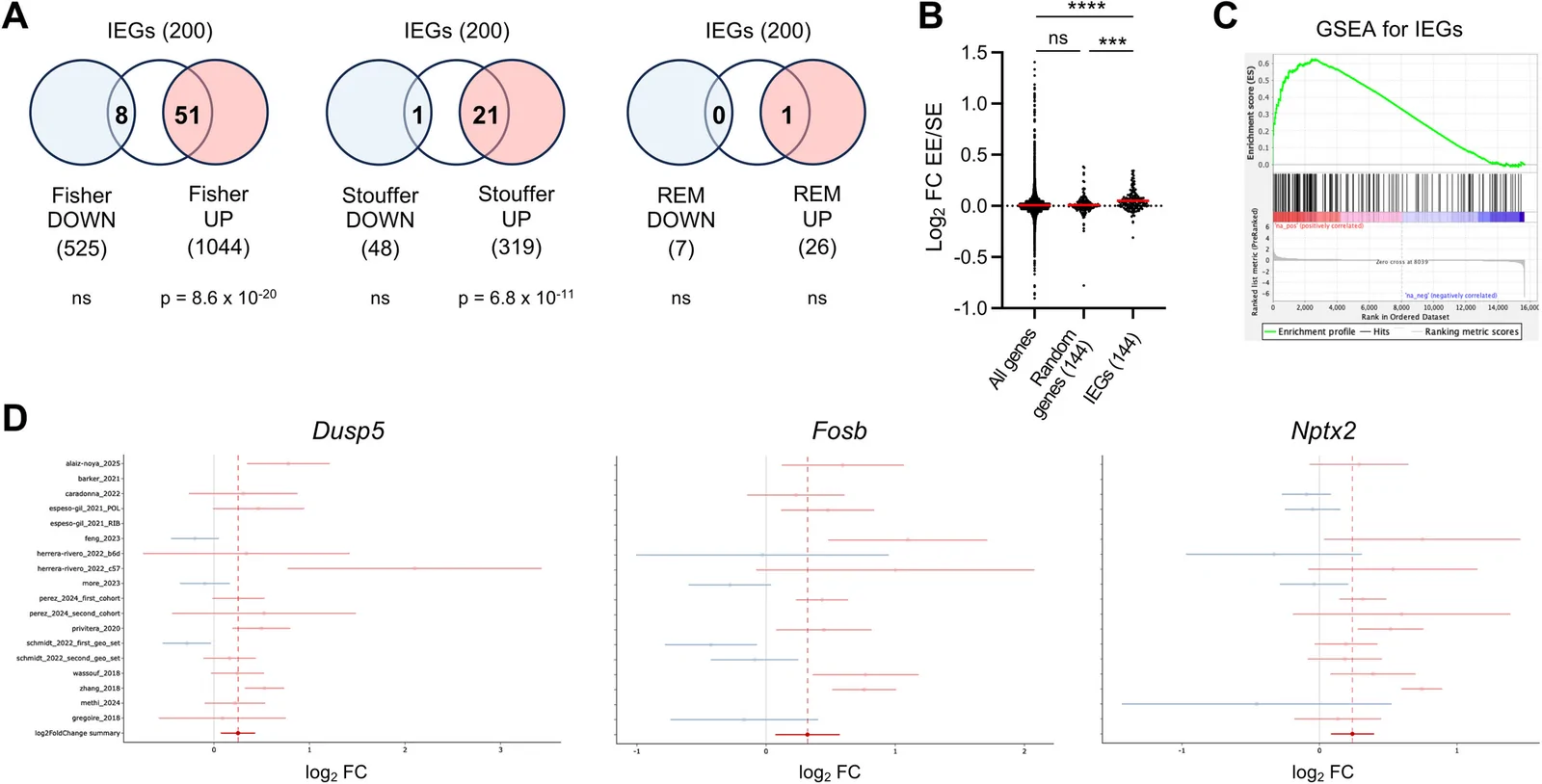
EE upregulates the activity-induced transcriptional program. **A** Venn diagrams displaying the overlap between IEGs as identified in [37] and significant upregulated and downregulated genes from meta-analysis of mouse RNA-seq datasets. A minimum of six datasets with detected gene expression values was required for a gene to be included. REM, Fisher and weighted Stouffer, adj *p*-value < 0.01. The hypergeometric probability for the intersections is shown. **B** Effect of EE on IEG expression levels compared to all genes and to a size-matched random gene subset. **C** GSEA of IEG gene set in REM meta-analysis of mouse datasets. A minimum of six datasets with detected gene expression values was required for a gene to be included. FDR q-value < 0.001. **D** Forest plots of *Dusp5*, *Fosb*, and *Nptx2* from REM meta-analysis of mouse datasets

To test our prediction that EE increases IEG expression in a more systematic manner, we crossed EE-dependent gene lists from our mouse meta-analysis with a previously published set of activity-induced genes in the mouse hippocampus [37]. For both Fisher’s and weighted Stouffer’s methods, we observed a highly significant overlap between IEGs and genes upregulated, but not downregulated, under EE conditions (Fig. 4A). As combined *p*-value methods primarily assess statistical significance rather than the direction of gene expression changes, we focused on REM results to evaluate the effect of EE on IEG regulation. Analysis of gene expression fold changes (Fig. 4B) and Gene Set Enrichment Analysis (GSEA) for the IEG gene set (Fig. 4C) confirmed a consistent and significant upregulation of activity-induced genes under EE across studies, as illustrated by canonical IEGs such as *Fosb*, *Dusp5*, and *Nptx2* (Fig. 4D). Remarkably, 10 out of the 12 DEGs consistently identified across microarray datasets were IEGs (Supp. Fig. S4B), further supporting our conclusion. Together, these results support the notion that EE promotes the activity-dependent transcriptional program, in agreement with its believed role in stimulating neuronal activity. This underscores the reliability of our meta-analysis strategy in capturing biologically meaningful molecular hallmarks of EE.

## Methods

### Literature Search

The systematic literature review and meta-analysis were undertaken according to Preferred Reporting Items for Systematic Reviews and Meta-Analyses (PRISMA) guidelines [85], and PRISMA checklist is shown in Supp. Table 12. PRISMA flow chart in Fig. 1A outlines our dataset collection and curation pipeline. We aimed to include transcriptomic studies published up to 10 February 2025 which met all the following four inclusion criteria: (1) employed EE paradigms, (2) used rodents as the animal model, (3) utilized genome-wide methods to measure gene expression, and (4) focused on brain regions or purified neuronal populations as tissue/cell-type of interest. To this aim, we queried the NCBI databases with the following search: (“environmental enrichment” OR “enriched environment” OR “EE model” OR “enriched environments”) AND (mouse OR mice OR murine OR rodent OR rat OR rats) AND (brain OR hippocampus OR cortex OR “central nervous system” OR CNS OR “nucleus accumbens” OR hippocampi OR hippocampal OR neurogenesis OR neurons) AND (“RNA-seq” OR “RNA sequencing” OR “transcriptome analysis” OR “transcriptomic profiling” OR “RNA-Seq data” OR “gene expression” OR “differential expression” OR transcriptomics OR microarray OR microarrays OR “molecular atlas” OR omics OR “multi-omics” OR “deep sequencing”). This search yielded a total of 322 peer-reviewed studies. In addition, we included a study from Angel Barco’s team, which has been recently accepted for publication [54].

We then manually screened the 323 studies to retain only those which fully satisfied the four inclusion criteria mentioned above. This selection process was conducted independently by three researchers through abstract review and, when necessary, access to the full text. Review articles were excluded. Concerning EE protocols (1), studies involving only a single exposure to an EE were excluded, because we considered this approach to represent a novelty exploration paradigm rather than an EE paradigm. Studies employing voluntary exercise without the object component were retained. We included papers which applied EE in the context of genetic, pharmacological or injury models of various brain pathologies; in such studies, we focused on EE-associated changes rather than in the disease-related changes. Regarding gene expression analysis methods (3), we included the studies that used RNA sequencing, both bulk and single-cell, or microarrays. One article employing SAGE-seq was also retained at this step since the data generated with this technique are consistent with RNA-seq analysis pipelines, although the study was ultimately excluded due to its read data being in an incompatible format with our analytical framework. In contrast, studies using non-genome-wide methods restricted to predefined gene sets, such as qPCR array and TaqMan arrays, were excluded. Four papers focused specifically on short RNA (sRNA) but data were available only for two of them, preventing the analysis of sRNA studies as a separate group. Consequently, we excluded those papers. Regarding brain regions and cell-types of interest (4), studies focused on the peripheral nervous system or on non-neuronal cell-types residing in the CNS were excluded. After this initial filtering, we retained 36 RNA-seq studies, including one using SAGE-seq, and 26 microarray studies.

Upon reviewing the full texts of the selected articles, four RNA-seq studies and seven microarray studies were excluded as they did not meet the predefined inclusion criteria. Then, we performed a second screen based on data availability. We excluded six RNA-seq articles due to the unavailability of FASTQ files, either publicly or upon request from the authors. Additionally, five RNA-seq papers were excluded because they did not include new experimental data but instead re-analyzed datasets already examined in other selected studies. We also excluded five microarray datasets since no DEG tables were published from these studies or no significant DEGs were detected. Furthermore, we removed another microarray study during data extraction since no fold change data were available. Following this second filtering step, based on data availability, we retained 20 RNA-seq and 13 microarray studies. The whole screening workflow is illustrated in Fig. 1A. A full list of the source papers included in the meta-analysis can be found in Supp. Table 1 and 2.

### Bias Assessment

The risk of bias within individual RNA-seq studies was evaluated across multiple domains, including subjective exclusion of outlier samples, sample pooling, absence of untreated or wild-type controls, and technical inconsistencies across samples. Each study was then assigned a risk-of-bias rating of “low,” “some concerns,” “moderate,” “serious,” or “high.” Potential sources of bias and the overall rating for each study are reported in Supp. Table 1.

### RNA-seq Dataset Meta-analysis

Raw sequencing data in FASTQ format were obtained from public repositories using accession identifiers provided in the respective publications and downloaded with fasterq-dump (v3.2.1). Sample files were renamed according to the metadata available in the corresponding repository. Quality control of the FASTQ files was performed using FastQC (v0.12.1) (https://www.bioinformatics.babraham.ac.uk/projects/fastqc/), and summary reports were generated with MultiQC (v1.28) [86]. Based on these results, filtering and trimming parameters were manually determined, and preprocessing was performed with fastp (v0.23.4) [87] using combinations of the flags −3, -x, -c, -p, and -f 1–3, depending on the QC outcomes.

Reads were mapped using RSEM (v1.3.1) [88] with STAR [89] as the aligner. Mouse datasets were aligned to the GRCm39 primary assembly with ENSEMBL GTF annotation v112, and rat datasets to the mRatBN7.2 primary assembly with ENSEMBL GTF annotation v113 [90]. Strand specificity was assessed using infer_experiment.py (v5.0.4) [91]. Mapping quality was evaluated with rsem-plot and by inspecting BAM files with samtools flagstat (v1.3.1) [92]. Transcript-level quantifications from isoforms results files were summarized to gene-level counts with tximport (v1.30.0) [93] using a tx2gene object generated from the same GTF file used for alignment. In one dataset, ComBat-seq (sva v3.50.0) [94] was applied to correct a strong batch effect, as recommended in correspondence with the study authors [68].

Differential expression analysis was performed with DESeq2 (v1.42.0) [95]. Variance-stabilized counts (vsn v3.70.0) [96] were explored via principal component analysis (PCA) using pcaExplorer (v2.28.0) [97] and hierarchical clustering heatmaps generated with pheatmap (v1.0.13) (https://github.com/raivokolde/pheatmap), applying Euclidean distance to either the top one hundred most variable genes or all genes. These analyses, combined with quality control observations, were used to identify and exclude outlier samples. The number of excluded samples for each dataset is reported in Supp. Table 1 under the columns “Number of samples” and “Remarks.” In some cases, clustering revealed the presence of multiple experimental batches. The two hundred most significant genes associated with the variable of interest, identified via *t*-tests, were represented in heatmaps, which can be visualized in the app. MA plots were generated using apeglm [98], ashr [99], and normal shrinkage methods and visualized with ggplot2 (v3.5.2) (https://ggplot2.tidyverse.org; H. Wickham. ggplot2: Elegant Graphics for Data Analysis. Springer-Verlag New York, 2016). The DESeq2 design formula was adapted according to the study complexity: datasets with three variables were split into two batches, those with two variables followed a 2 × 2 design for meta-analysis, and those with a single variable employed a simple design. For downstream meta-analysis, in addition to the two-sided tests for generating results employed in effect size combination methods, one-sided Wald test p-values were generated for use in *p*-value combination methods. Differentially expressed genes were defined as those with an adjusted *p*-value < 0.05. DEG identification was robust to the manual curation process (Supp. Fig. S2D, E). Gene names and biotypes were annotated using EnsDb.Mmusculus.v79 and EnsDb.Rnorvegicus.v79 [100].

Log₂ fold change combination for meta-analysis was performed with MetaVolcanoR (v1.16.0) (Prada C, Lima D, Nakaya H, 2024), with custom modifications to the rem_mv, plot_rem and draw_forest functions for visualization. The resulting metaresult object was filtered to retain only genes represented in at least six contributing studies for mouse datasets and at least two for rat datasets, after which *p*-values were adjusted using the Benjamini–Hochberg (B-H) method. The rma function from the metafor package (v4.8.0) (https://cran.r-project.org/web/packages/metafor/index.html) was used to extract heterogeneity metrics from the random-effects model and to conduct meta-regression analyses. Moderators were specified using the formula ~ Sex + EE_duration_group + CNS_region_group + Pathology_group for the full model and ~ EE_duration_group for the reduced model.

MetaP (v1.12) (https://cran.r-project.org/web/packages/metap/index.html) was used to combine *p*-values via Fisher’s method (sumlog) and the weighted Stouffer method (sumz). For the latter, an epsilon correction was applied to weights equal to 1 to ensure all values were below 1. Filtering and B-H adjustment were applied as in the random-effects model analysis.

For data visualization, interactive plots were generated using plotly (v4.10.4) (Sievert C, 2020; https://plotly-r.com), and batch effects were corrected with ComBat from the sva package (v3.50.0) [101]. Color schemes in principal component analysis plots were defined using RColorBrewer (v1.1.3) (Neuwirth E, 2022; https://CRAN.R-project.org/package=RColorBrewer). To enable cross-species integration, rat genes were mapped to their mouse orthologs using biomaRt (v2.58.2) [102], querying the rnorvegicus_gene_ensembl dataset for the attributes “ensembl_gene_id” and “mmusculus_homolog_ensembl_gene.” Gene identifiers were subsequently converted to gene symbols using org.Mm.eg.db or org.Rn.eg.db (Carlson M, 2023).

### Microarray Dataset Integration

Gene symbols and fold changes were extracted from microarray studies. Gene symbols were compared with the NCBI database and microarray annotation data. Old gene symbols were updated by using the biomaRt- (v 2.65.0) [102], mygene- (v 1.44.0) (Mark, Thompson, Afrasiabi, Wu (2025). https://bioconductor.org/packages/mygene), and annotate (v 1.86.1) (Gentry J (2025). https://bioconductor.org/packages/annotate) R packages. Fold changes were log_2_-transformed to symmetrize effect sizes around zero, and a combined log_2_ FC was calculated as the arithmetic mean of individual log_2_ FC values.

### Gene Ontology (GO) and Gene Network Analysis

Enrichment analysis in the Shiny app was performed using clusterProfiler (v4.10.1) together with the annotation packages org.Mm.eg.db or org.Rn.eg.db. Genes annotated with ENSEMBL IDs were first converted to Entrez IDs prior to analysis. All three Gene Ontology domains provided by the package were included. For the universe parameter, all genes present in the result matrix were used, with the final background set defined as the intersection between the database and the genes in the matrix, consistent with the package’s default behavior. The CellMarker database for mouse was downloaded from bio-bigdata.center in January 2025. For Gene Set Enrichment Analysis (GSEA) [103], the complete gene list from REM analysis was ranked by the product of the sign of the summary log2 fold change and the negative logarithm of the summary p-value. A minimum of six datasets with detected values was required for a gene to be included in the ranked list. For GSEA and EE/SE fold change analysis of IEGs, the 200 genes most strongly upregulated in the hippocampus after kainate treatment were used [37], among which 144 were detected in at least 6 datasets. For SynGO analysis [104], mouse genes were converted to their human homologues with SynGO ID conversion tool before GO analysis. Protein network analysis was performed with STRING [105] on the significant upregulated genes found from mouse studies with Fisher’s method (adj *p*-value = 0.01). Only the physical subnetwork was taken into account, using textmining, experiments, and databases as interaction sources (minimum required interaction score = 0.4). The resulting network was exported to Cytoscape [106] for visualization.

## Discussion

Since the widespread adoption of microarray technology in the 2000 s and next-generation sequencing in the 2010 s, numerous studies have investigated the impact of EE on the brain transcriptome [7]. However, there has been limited effort to integrate these datasets and determine consistent gene expression signatures associated with EE. To address this gap, we conducted what is, to our knowledge, the first meta-analysis of brain transcriptomic datasets from rodents exposed to EE.

Across our broad dataset collection (33 studies), a direct intersection of EE-induced DEGs from individual datasets revealed only limited overlap. This inconsistency likely reflects both methodological and biological factors. Differences in experimental design, such as sex, age, strain, group size, duration of EE exposure, object characteristics (e.g., shape, texture, size), object replacement frequency, and the presence or absence of running wheels, likely influenced transcriptional outcomes, and argue for standardization of EE protocols to improve reproducibility. In addition, prolonged EE exposure can trigger neuroadaptive gene expression responses which may differ between individuals [21, 107], introducing additional variability even across animals housed under identical conditions. Biological sources of variability also include the specific brain region analyzed in each study, and the high cell-type heterogeneity of neural tissue, which may mask cell-type-specific responses.

To overcome study heterogeneity and uncover gene expression signatures consistently associated with EE, we implemented three meta-analysis approaches for RNA-seq datasets: Fisher’s and weighted Stouffer’s methods, which combine *p*-values, and the random effect model, which accounts for the magnitude and direction of gene expression changes. These methods differ in their assumptions and statistical properties [71]. Indeed, Fisher’s analysis found the largest number of significant genes, consistent with its greater sensitivity to small *p*-values compared to the more conservative Stouffer’s method [72]. Regarding microarray studies, the lack of available raw data restricted their integration to comparisons of DEG lists.

Our meta-analysis of mouse RNA-seq datasets identified sets of genes and pathways consistently modulated by EE. A key finding was the enrichment of synaptic genes. Despite most source datasets were obtained from bulk tissue and thus lacked cell-type resolution, the pronounced overrepresentation of synaptic GO terms clearly points to a major effect of EE on neuronal populations. The involvement of structural components of the postsynaptic density (PSD), core subunits and regulators of the synaptic vesicle machinery, ion channels, and synaptic adhesion proteins supports the growing consensus that EE influences neuronal and spine morphology and synaptic function. Several studies have shown that EE increases spine density, dendritic length, and dendritic complexity in hippocampal and cortical regions [17, 22–24]. Recordings from hippocampal neurons revealed that EE increases cell excitability, specifically enhances excitatory synaptic transmission in DG granule neurons, and facilitates long-term potentiation (LTP) in CA1 pyramidal neurons [5, 108–110]. While experimental manipulation of the identified genes and pathways is required to infer causality, the transcriptomic changes revealed by our meta-analysis likely act together to support the morphological and functional synaptic adaptation underlying EE beneficial effects.

Notably, Gene Ontology (GO) terms related to synapses were strongly overrepresented among both upregulated and downregulated genes. This pattern could, in principle, arise from the properties of Fisher’s method, which emphasizes small p-values from individual studies even when others show weak or no effects, potentially leading to the same gene being classified as both upregulated and downregulated. However, we found that only 22 genes were modulated in both directions, representing a median of 3.37% of the genes within each of the top enriched GO categories. Thus, the shared enrichment of synaptic GO terms among up- and downregulated genes is unlikely to be an artifact of the meta-analysis method itself, but instead genuinely reflects the complex and widespread remodeling of the synaptic transcriptome induced by environmental enrichment.

We also found that EE upregulates the activity-dependent transcriptional program, a finding that was also supported by microarray studies. This evidence supports, at the gene expression level, the general notion that enhanced sensory, cognitive, motor, and social stimulation triggers neuronal activation across cortical and hippocampal regions [6]. Whereas other studies had shown IEG upregulation under EE [45, 46, 54], our meta-analysis generalizes this conclusion across tens of datasets. Notably, among the significantly affected IEGs, we identified multiple FOS and JUN family members which form the activity-induced AP-1 transcriptional complex. AP-1 is a master regulator of the activity-dependent transcriptional program [83], controls synaptic gene expression [84, 111], and has been proposed to mediate the cognitive benefits of EE [54]. In light of these findings, AP-1 likely plays a central role in the modulation of synaptic genes by EE revealed by our meta-analysis. Gene regulatory network analysis could potentially provide further insights into the transcriptional regulatory layers underlying the observed gene expression changes, but attempting it would require adapting the approach to each study and is therefore beyond the scope of this work. Altogether, these results demonstrate the power of our systematic approach in uncovering consistent molecular signatures of EE across diverse experimental conditions.

To facilitate biological interpretation of the RNA-seq meta-analysis and enable data interrogation tailored to specific research questions, we developed mEEtaBrain, an interactive online app to explore and compare results across methods. This tool is made publicly available to all of the research community so that any researcher interested in a subset of the studies we have analyzed can get their own lists of genes that would meet their own significance criteria. We hope that this will greatly improve the impact of our analysis on the scientific community.

The intersection of EE-dependent genes between mice and rats was very small. We believe that the discrepancy might more likely originate from the quality and quantity of rat datasets rather than from genuine inter-species biological differences. Importantly, in 5 of the 6 rat studies [55–59], the authors examined the effect of EE in the context of severe stressors such as cocaine administration and lead exposure, or using models of brain conditions, thereby introducing a substantial confounding factor in the comparison between SE and EE. Also, the limited number of rat studies likely reduced statistical power, resulting in much fewer significant genes compared to the mouse meta-analysis and contributing to the modest inter-species overlap. Furthermore, the large majority of mouse datasets were obtained from cortex or hippocampus, which predominantly contain glutamatergic neurons, whereas half of rat studies were conducted on striatum, almost entirely composed of GABAergic neurons. This discrepancy in the brain regions examined, together with the other factors mentioned above, likely contributes to the apparent lack of overlap between the two species. Finally, reported differences in cognitive performance and stress resilience between mice and rats [112, 113] could also influence how EE affects brain transcriptomes in each species.

This study presents some limitations. Substantial heterogeneity in experimental design, EE protocol, tissue of interest, and neuronal populations across the source studies likely increased variability in the meta-analysis outcomes. Nevertheless, our heterogeneity assessment and meta-regression analyses indicate that the principal transcriptional signatures identified in this study are not driven by substantial between-study heterogeneity, supporting the robustness of the core findings despite methodological diversity. Likewise, although brain region did not emerge as a major source of heterogeneity in the meta-regression analyses, these results should be interpreted with caution, as several brain regions and neuronal populations were represented by only a limited number of datasets. Consequently, we cannot exclude the possibility that region- or cell type-specific transcriptional responses to EE were masked in the present analysis. The use of stressors or disease models in some studies introduced a confounding factor and limited reliable interspecies comparison. Raw data were unavailable for most microarray studies, preventing the conduct a proper meta-analysis. Overall, the studies included in the meta-analysis exhibited a low to moderate risk of bias.

In addition to transcriptomic analysis, several works have examined the impact of EE on chromatin profiles, including histone acetylation, histone and DNA methylation, and chromatin accessibility [46, 49, 51, 54]. Future research should aim to determine core EE-dependent epigenetic modifications across studies using a systematic approach, and relate these changes to the gene expression patterns uncovered in our analysis. Pinpointing critical chromatin marks that regulate EE-responsive genes could open new opportunities to modulate their expression, and support mechanistic studies to identify potential therapeutic targets able to mimic or strengthen the cognitive benefits of EE.

In conclusion, our meta-analysis extracted core gene expression signatures of EE across dozens of studies. To make these findings accessible, we created an intuitive web app for navigating genes and pathways influenced by EE. Importantly, the mEEtaBrain app also makes it possible for the community to use this interface to ask their own questions by adjusting the subset of studies that can be taken into account and the required significance level. We believe that this resource will advance understanding of the molecular basis of EE-driven behavioral effects, and foster new hypotheses within the neuroscience community.

## Supplementary Information

Below is the link to the electronic supplementary material.

Supplementary Material File 1 (XLSX 17.3 KB)

Supplementary Material File 2 (XLSX 14.5 KB)

Supplementary Material File 3 (XLSX 15.3 KB)

Supplementary Material File 4 (XLSX 12.6 KB)

Supplementary Material File 5 (XLSX 70.3 KB)

Supplementary Material File 6 (XLSX 23.8 KB)

Supplementary Material File 7 (XLSX 12.5 KB)

Supplementary Material File 8 (XLSX 9.82 KB)

Supplementary Material File 9 (XLSX 62.6 KB)

Supplementary Material File 10 (XLSX 54.7 KB)

Supplementary Material File 11 (XLSX 72.0 KB)

Supplementary Material File 12 (DOCX 31.5 KB)

Fig. 5 Low Resolution Supplementary File 13 (PNG 221 KB)

High Resolution Supplementary File 13 (TIF 2.42 MB)

Fig. 6 Low Resolution Supplementary File 14 (PNG 308 KB)

High Resolution Supplementary File 14 (TIF 3.46 MB) 

Fig. 7 Low Resolution Supplementary File 15 (PNG 544 KB)

High Resolution Supplementary File 15 (TIF 4.40 MB)

Fig. 8 Low Resolution Supplementary File 16 (PNG 242 KB)

High Resolution Supplementary File 16 (TIF 2.55 MB)

Fig. 9 Low Resolution Supplementary File 17 (PNG 218 KB)

High Resolution Supplementary File 17 (TIF 2.18 MB)

Supplementary Material File 18 (ZIP 48.1 KB)

Supplementary Material File 19 (DOCX 21.3 KB)

## Data Availability

All data analyzed in this study were obtained from publicly available databases, with the exception of the dataset from Utsunomiya et al., which is not publicly available and was provided directly by the authors upon request. Detailed information on each dataset is available in Supplementary Tables 1 and 2, and all publicly accessible data can be retrieved through the respective platforms. Scripts used for preprocessing raw FASTQ files, including Fastp trimming and RSEM quantification, are available in Supplementary File 1. All pre-processed datasets used in the mEEtaBrain application (http://regulomics.mimuw.edu.pl/mEEtaBrain/), representing the results of DESeq2 differential expression analyses, are publicly available via the Zenodo repository at https://zenodo.org/records/21355440. A video tutorial demonstrating the use of the mEEtaBrain application is available at https://www.youtube.com/watch?v=neqPSVcyRwA.

## Declarations

### Generative AI in the Writing Process

During the preparation of this work, the authors used ChatGPT to revise English grammar and usage, as well as to assist with software development tasks as a coding copilot. After using this tool, the authors reviewed and edited the content as needed and take full responsibility for the content of the publication.
