# Supplementary material for: Systematic Review and Transcriptomic Meta-analysis of Environmental Enrichment Reveal Core Molecular Programs of Brain Plasticity
Source: Mol Neurobiol. 2026 Aug 26;63(1):857. doi: 10.1007/s12035-026-06133-y (PMC13506623; doi:10.1007/s12035-026-06133-y)
Supplement: Supplementary file 2 [file 12035_2026_6133_MOESM19_ESM.docx]

**SUPPLEMENTARY MATERIAL**

- **Supplementary Figures S1-S5**

- Supplementary Tables:

**Supp. Table 1.** Summary of mouse and rat RNA-seq datasets collected for the meta-analysis.

**Supp. Table 2.** Summary of mouse and rat microarray datasets collected for the meta-analysis.

**Supp. Table 3.** EE conditions in the mouse and rat RNA-seq datasets.

**Supp. Table 4.** EE conditions in the mouse and rat microarray datasets.

**Supp. Table 5.** Significant genes in the Fisher’s method meta-analysis of mouse RNA-seq datasets.

**Supp. Table 6.** Significant genes in the weighted Stouffer’s method meta-analysis of mouse RNA-seq datasets.

**Supp. Table 7.** Significant genes in the REM meta-analysis of mouse RNA-seq datasets.

**Supp. Table 8.** Shared DEGs across mouse microarray datasets.

**Supp. Table 9.** GO enrichment analysis of significant genes after Fisher’s method meta-analysis of mouse RNA-seq datasets.

**Supp. Table 10.** SynGO analysis of significant genes after Fisher’s method meta-analysis of mouse RNA-seq datasets.

**Supp. Table 11.** STRING interaction network of significant upregulated genes after Fisher’s method meta-analysis of mouse RNA-seq datasets.

**Supp. Table 12.** PRISMA 2020 Main Checklist.

- **Supplementary File 1**. ZIP file containing the scripts for the raw FASTQ files pre-processing using Fastp and RSEM.

**Supplementary Figure S1 related to Figure 2. Overview of rat RNA-seq studies and meta-analysis. A.** Heatmap showing the number of detected genes shared between pairs of rat RNA-seq studies. **B.** Heatmap showing the number of DEGs shared between pairs of rat RNA-seq studies. **C.** PCA of rat RNA-seq datasets. EE, Enriched Environment (red); SE, Standard Environment (blue); N = 36 (EE), 37 (SE). **D.** Number of genes for which meta-analysis statistics were computed plotted against the minimum number of studies in which each gene was detected.

**Supplementary Figure S2 related to Figure 2. Robustness assessment of the meta-analysis approaches.** **A-C.** The leave-one-study-out analyses show that the median change in the number or proportion of genes affected by the removal of a single study is typically near 0 in mouse and mostly near 0 in rat, except for downregulated genes identified with the Fisher method, largely due to the small number of genes reported in rat. **D, E.** Comparison of DEG number between manually curated and original data indicate mostly negligible changes, with the exception of the Perez study, which used two separate animal cohorts without clearly specifying this in the original analysis. The substantial change in detected genes for this study reflects our reinstatement of this cohort separation for the analysis, as described in the paper.

**Supplementary Figure S3 related to Figure 2. Assessment of heterogeneity and moderator effects in the meta-analysis.**

**A, B.** The distribution of the I^2^ statistics for EE effect sizes estimated using REM are shown for mouse (A) and rat genes (B). **C, D.** Cochran’s QE test p-values are plotted against the corresponding I^2^ values for mouse (C) and rat genes (D). Genes identified as significant in the REM analysis are highlighted in red. Significant Cochran’s QE test p-values are observed predominantly among genes with I^2^ values above 60 %, whereas genes significant in the REM analysis show little overlap with genes exhibiting significant heterogeneity or high I^2^ values. **E.** The estimated coefficients of the moderator variables included in the meta-regression model for mouse studies are shown. Rat studies were not analyzed because of insufficient data. Coefficients were generally centred around zero, including for genes significant in the REM analysis, which are highlighted in red. **F.** The p-values of the moderator variables included in the meta-regression model for mouse studies are shown, with significant outliers observed primarily for moderators related to EE duration. **G.** REM effect sizes are stratified according to whether the EE duration moderator coefficient was significant or non-significant in reduced model, with genes coloured according to their significance in Fisher’s analysis. Boxplot outliers are indicated by grey points; the vast majority overlap with genes identified as significant by Fisher’s method, while the remaining visible grey points represent outlier genes not detected in Fisher’s analysis.

**Supplementary Figure S4 related to Figure 2. Shared DEGs across microarray studies. A.** Heatmap showing the number of DEGs shared between pairs of microarray studies. **B.** List of DEGs shared by at least three studies, and their combined log_2_ FC. IEGs are indicated in red. More detailed information about the DEGs can be found in **Supp. Table 8**. **C.** Venn diagrams displaying the overlap among gene lists identified with the different meta-analysis methods applied to mouse RNA-seq datasets. A minimum of six datasets with detected values was required for a gene to be included. REM, Fisher and weighted Stouffer, adj p-value < 0.01. Numbers in parenthesis indicate how many genes are also differentially expressed in the combined microarray analysis.

**Supplementary Figure S5 related to Figure 3. GO enrichment analysis for Fisher’s method upregulated and downregulated genes. A.** Bubble plots illustrating the 15 most significant GO terms for Molecular Function are shown. The bubble size and colour indicate the number of genes associated to the term and the adjusted p-value, respectively.
